# Prevalence and Correlates of Bacterial Vaginosis in Different Sub-Populations of Women in Sub-Saharan Africa: A Cross-Sectional Study

**DOI:** 10.1371/journal.pone.0109670

**Published:** 2014-10-07

**Authors:** Vicky Jespers, Tania Crucitti, Joris Menten, Rita Verhelst, Mary Mwaura, Kishor Mandaliya, Gilles F. Ndayisaba, Sinead Delany-Moretlwe, Hans Verstraelen, Liselotte Hardy, Anne Buvé, Janneke van de Wijgert, for the Vaginal Biomarkers Study Group

**Affiliations:** 1 Unit of Epidemiology and Control of HIV/STD, Department of Public Health, Institute of Tropical Medicine, Antwerp, Belgium; 2 HIV/STI Reference Laboratory, Department of Clinical Sciences, Institute of Tropical Medicine, Antwerp, Belgium; 3 Clinical Trials Unit, Department of Clinical Sciences, Institute of Tropical Medicine, Antwerp, Belgium; 4 International Centre for Reproductive Health (ICRH), Ghent University, Ghent, Belgium; 5 ICRH Kenya, Mombasa, Kenya; 6 Rinda Ubuzima, Kigali, Rwanda; 7 Wits Reproductive Health & HIV Institute, University of Witwatersrand, Johannesburg, South Africa; 8 Department of Obstetrics and Gynecology, Faculty of Medicine and Health Sciences, Ghent University, Ghent, Belgium; 9 Department of Clinical Infection, Microbiology and Immunology, Institute of Infection and Global Health, University of Liverpool, Liverpool, United Kingdom, and AMC-CPCD, Amsterdam, The Netherlands; Fred Hutchinson Cancer Center, United States of America

## Abstract

**Background:**

Clinical development of vaginally applied products aimed at reducing the transmission of HIV and other sexually transmitted infections, has highlighted the need for a better characterisation of the vaginal environment. We set out to characterise the vaginal environment in women in different settings in sub-Saharan Africa.

**Methods:**

A longitudinal study was conducted in Kenya, Rwanda and South-Africa. Women were recruited into pre-defined study groups including adult, non-pregnant, HIV-negative women; pregnant women; adolescent girls; HIV-negative women engaging in vaginal practices; female sex workers; and HIV-positive women. Consenting women were interviewed and underwent a pelvic exam. Samples of vaginal fluid and a blood sample were taken and tested for bacterial vaginosis (BV), HIV and other reproductive tract infections (RTIs). This paper presents the cross-sectional analyses of BV Nugent scores and RTI prevalence and correlates at the screening and the enrolment visit.

**Results:**

At the screening visit 38% of women had BV defined as a Nugent score of 7–10, and 64% had more than one RTI (*N. gonorrhoea*, *C. trachomatis*, *T. vaginalis*, syphilis) and/or *Candida*. At screening the likelihood of BV was lower in women using progestin-only contraception and higher in women with more than one RTI. At enrolment, BV scores were significantly associated with the presence of prostate specific antigen (PSA) in the vaginal fluid and with being a self-acknowledged sex worker. Further, sex workers were more likely to have incident BV by Nugent score at enrolment.

**Conclusions:**

Our study confirmed some of the correlates of BV that have been previously reported but the most salient finding was the association between BV and the presence of PSA in the vaginal fluid which is suggestive of recent unprotected sexual intercourse.

## Introduction

UNAIDS estimated that in 2012 0.5% and 0.3% of women and men aged 15–24 years were living with HIV globally [1]. There were, however, important regional differences with the highest prevalence rates reported in sub-Saharan Africa, 2.5% and 1.2% among young women and men, respectively. The high vulnerability to HIV acquisition of young women in sub-Saharan Africa compared to young men can be explained by a combination of behavioural factors including patterns of age mixing; socio-cultural factors and imbalances in gender power relations; and biological factors. Among the latter, infection with herpes simplex virus-2 (HSV-2) and deviations from the normal lactobacilli dominated vaginal microbiota take a prominent place [2]–[5]. For instance, in a cohort of HIV uninfected women in Uganda and Zimbabwe it was estimated that 50% of incident HIV infections were attributable to HSV-2 infection, 17% to bacterial vaginosis (BV) and 12% to intermediate microbiota as defined by Nugent score [5].

Novel interventions addressing the vulnerability of young women to HIV and other sexually transmitted infections (STIs) are urgently needed. These will include vaginally applied products, such as medicinal products containing antiretroviral drugs, and ‘multipurpose’ devices releasing antimicrobial compounds and hormones to prevent reproductive tract infections and pregnancy [6]. These innovative methods should also restore and/or maintain a healthy vaginal environment with its innate defence mechanisms. During clinical development of vaginally applied products, it became clear that the vaginal environment needs to be better characterised in order to establish a ‘baseline’ which can be used to evaluate the effects of candidate vaginal products. Better characterisation is not only needed in the ‘general population’ at low or average risk of HIV, it is also needed in subpopulations that are often targeted for reproductive health interventions, such as adolescent girls, pregnant women, HIV-positive women, and women at high risk of HIV infection.

In the research project entitled “Characterisation of novel microbicide safety biomarkers in East and South Africa”, we set out to characterise the vaginal environment of a representative sample of HIV negative adult women at low risk for HIV. The vaginal environment was also studied in smaller sub-populations of women in Kenya, South-Africa and Rwanda. The main aim of the project was to characterise the vaginal microbiota, to describe the mucosal immune responses in the vagina and to assess changes over time. In this paper, we present the socio-demographic and behavioural characteristics of the study populations, the prevalence of BV and reproductive tract infections (RTIs) and correlates of BV and RTIs at screening and at enrolment in a cross-sectional analysis.

## Methods

### Study design and population

Women were screened and enrolled in 2010–2011 at three study sites: the International Centre for Reproductive Health Kenya (ICRHK), Mombasa, Kenya; the Wits Reproductive Health and HIV Institute (WrHI), Johannesburg, South Africa (SA); and the non-governmental organisation Rinda Ubuzima (RU), Kigali, Rwanda. Although this paper focuses on the cross-sectional data from the screening and the enrolment visit, participants were followed up for approximately eight months per person. All women, eligible or not, were offered diagnostic testing for RTIs at the screening visit. The results were evaluated with the participants two weeks later when treatment was provided. Enrolment of eligible participants was then scheduled after the next menses (approximately day 9 of the menstrual cycle; window of 2 days). A further description of the full study schedule is available in Methods S1.

Women were recruited into per protocol predefined study groups as follows: adult, non-pregnant, HIV-negative women at average risk of HIV (referred to in this paper as the reference group); pregnant women (up to 14 weeks gestation); adolescent girls (16–17 years); HIV-negative women engaging in vaginal practices; self-acknowledged female sex workers (FSW); and HIV-positive women on antiretroviral treatment. The women in the reference group and the pregnant women were recruited from family planning clinics, ‘women's groups’ and antenatal clinics in Mombasa County and Johannesburg. Adolescent girls were recruited at youth centres and youth-friendly family planning services in Mombasa and Johannesburg. Women engaging in vaginal practices were recruited in Johannesburg only. These women used substances (cloth/lemon juice/detergents) other than water and/or fingers to clean, dry or tighten the vagina on a regular basis. FSW and HIV-positive women were recruited in Kigali from the sex worker community, using community mobilisers and from public HIV treatment clinics, respectively.

Participants were eligible for inclusion in the study if they were in good physical and mental health; able and willing to participate in the study as required by the protocol; able and willing to give written informed consent (and written assent for minors), including written parental/guardian consent for adolescents; and HIV-negative unless confirmed HIV-positive for inclusion in the HIV-positive women group. Pregnant women were included in the study if they were up to 14 weeks gestational age as defined by abdominal ultrasound. HIV-positive women were included if they had been on antiretroviral treatment for at least 6 months, were asymptomatic and had a CD4 count of more than 350 cells/µl. Participants meeting one or more of the following criteria were excluded from the study: history of hysterectomy and other genital tract surgery in the three months prior to the screening visit; never having had penetrative vaginal intercourse; enrolled in HIV prevention trials involving investigational products; confirmed internal and/or external genital warts at screening and/or enrolment; breastfeeding or less than 6 months post-partum at the time of enrolment; or pregnant (unless for inclusion in the pregnant women group).

### Study procedures

Women who expressed an interest in participating in the study were asked twice for their written informed consent: first, to be screened for eligibility for study participation and second, to participate prior to enrolment. Diagnostic testing was performed at the screening visit only and consisted of the following tests: HIV, HSV-2, syphilis, *Neisseria gonorrhoea, Chlamydia trachomatis*, *Trichomonas vaginalis*, urinary tract infection (UTI), pregnancy, cervical dysplasia screening by Pap smear, BV based on the Amsel criteria, and vaginal candidiasis. Treatment to participants was provided according to local standard operational procedures based on national guidelines. Firstly, symptomatic women with positive microscopy results for trichomoniasis, BV (by Amsel criteria), and candidiasis were treated at the screening visit and secondly, women testing positive for syphilis, trichomoniasis, *N. gonorrhoea*, and/or *C. trachomatis* received treatment at the result visit. STI/RTI testing was not routinely repeated at enrolment but was available for women presenting with symptoms. Women received voluntary HIV counselling and testing according to national guidelines, and condoms were provided free-of-charge at all visits. Participants who refused to know their HIV status were ineligible. Ineligible participants were offered diagnostic testing for vaginal infections and screening for cervical cancer. Curable RTIs and UTIs were treated at the study clinics, while women with abnormal Pap smear, HIV-positive women and pregnant women were referred to appropriate local public clinics.

At both visits, women were interviewed about their general and sexual health. A physical and speculum examination was carried out, and blood and vaginal/endocervical samples were collected. Ascertainment of BV for research purposes was performed with Gram stain Nugent scoring by staff at the Institute of Tropical Medicine in Antwerp for the screening and enrolment visits. At enrolment a specific set of specimens for the ‘characterisation of the vagina’ was obtained from each participant including the Gram stain for BV, a vaginal pH measurement and prostate specific antigen (PSA).

### Laboratory methods

Women in all sites were tested for HIV using a serial testing algorithm including rapid simple tests. All sites used the Determine HIV-1/2 (Abbott Diagnostic Division, Hoofddorp, The Netherlands) as a first test. In case this was reactive, the specimen was tested using the SD Bioline (Standard Diagnostics Inc., Kyonggi-do, South Korea) in Kigali and Mombasa or the Uni-Gold HIV (Trinity, Berkeley Heights, New Jersey, USA), in Johannesburg. The latter test was used as a tiebreaker test in Kigali and Mombasa. In Johannesburg an ELISA 4th generation assay (Abbott Diagnostic Division, Hoofddorp, The Netherlands) was employed. HSV-2 antibodies were detected using a HSV-2 IgG ELISA (Kalon Biologicals, Aldershot, United Kingdom). Syphilis serology was assessed with RPR (Spinreact (Spinreact Reactivos, Girona, Spain)) in Kigali, BD Macro-Vue (Becton, Dickinson and Co, Maryland, USA) in Mombasa and Johannesburg, followed by TPPA (Serodia-TP.PA (Fujirebio Diagnostics, Pennsylvania, USA.)). Endocervical swabs were used to test for *C. trachomatis* and *N. gonorrhoeae* by real-time PCR (Abbott Realtime CT/NG (Abbott Diagnostic Division, Hoofddorp, The Netherlands)), Diagenode CT/NG RT_PCR (Liège, Belgium), BD ProbeTec CT/NG (Becton, Dickinson and Co, Maryland, USA) in Rwanda, Mombasa and Johannesburg, respectively.

A microscopic assessment of a wet mount was performed to visualise *Candida* cell budding and hyphae, *T. vaginalis* and clue cells. *T. vaginalis* was cultured using InPouch culture pouches with assessments at 3 and 5 days after inoculation (Biomed Diagnostics, Orlando, USA). BV was diagnosed by Amsel criteria for diagnosis and immediate treatment. For the research project and data analysis Nugent scoring of Gram stained smears was performed off-site by 2 experienced laboratory technicians at the Institute of Tropical Medicine. A score 0–3 was normal (BV-negative); a score 4–6 meant intermediate microbiota; and score 7–10 was BV positive. Urine was tested for pregnancy by rapid hCG test and for infection using a urinalysis dipstick. The vaginal pH was measured using pH 3.6–6.1 paper strips pressed against the vaginal wall during the pelvic examination (Macherey-Nagel pH Fix 3.6–6.1, Düren, Germany). Vaginal swab material was eluted and tested for the presence of PSA using the Seratec PSA semiquant assay (Seratec Diagnostica, Göttingen, Germany).

### Data analysis

We describe here the prevalence of BV and RTIs and correlates of BV and RTIs among women who were enrolled. This was done separately for the screening visit and for the enrolment visit. The reason for this is that women, who were found to have BV or a treatable RTI at screening, were treated and may have been cured by the time they were enrolled into the study. For the correlates of BV and RTI at screening, data on sexual behaviour were used that were collected at the enrolment visit. For the correlates of BV at the enrolment visit, the RTI status at the screening visit was used.

Study population characteristics and data on reproductive health are described as means with ranges for continuous variables and counts and percentages for categorical variables. A composite variable was a-priori defined for sexual risk taking. Women were considered as “low risk” if they had less than 2 partners in the last year *and* in the last 3 months did not have any partner who had multiple partners *and* were over 14 years old at sexual debut. “Medium risk” was defined as 2 partners in the last year *or* in the last 3 months at least one sexual partner who had multiple partners. Women were considered at “high risk” if they were sex workers *or* had at least 3 partners in the last year *or* had at least one sexual partner with HIV in the last 3 months *or* had their sexual debut before age 15.

No formal statistical testing of between-group differences in baseline characteristics was performed. When determining correlates of BV, vaginal candidiasis, and RTIs using logistic regression modelling, a list of potential correlates was defined a-priori based on clinical and biological relevance. The variable “recent antibiotic use” was defined as recently started systemic antibiotics that had been used in the 14 days before the enrolment visit. We did not include antibiotic treatment for opportunistic infections because of collinearity with HIV-status. When more than one treatment was initiated in the 14 days prior to enrollment the longest treatment was chosen. Bivariable associations were assessed between each of the potential correlates and BV (defined as a Nugent score of 7–10 versus Nugent score 0–6) at the screening and enrolment visits, as well as vaginal candidiasis and RTIs at the screening visit. Factors significant at p≤0.100 were included in multivariable models which were simplified through stepwise deletion until all factors were independently associated with BV, vaginal candidiasis or RTIs at p≤0.050 [7]. The final model was summarised using adjusted odds-ratio, 95% confidence intervals and p-values. The dataset is available as Table S1.

### Ethics statement

All interviews were conducted by a qualified person identified by the Principal Investigator and done in the language chosen by the participants. Written information and consent forms in the local language were provided to the women or legally authorized representatives for their review. After the interview, the participants and, in case they were of minor age/not emancipated (age below 18 in South Africa and Kenya and below 21 in Rwanda), the parents or guardians were asked to confirm their willingness to participate in the study by signing (or thumb-printing whenever they are illiterate) the consent form. The protocol was approved by the Kenyatta National Hospital Ethical Review Committee, Kenya; the Human Research Ethics Committee (Medical), University of the Witwatersrand, SA; the Rwanda National Ethics Committee, Rwanda; the Institutional Review Board of the Institute of Tropical Medicine in Antwerp, Ghent University, and the University Teaching Hospital in Antwerp, Belgium. In addition the study was approved by the National Council on Science and Technology in Kenya; the SA Department of Health; and the National AIDS Control Commission in Rwanda.

## Results

**Figure 1:**
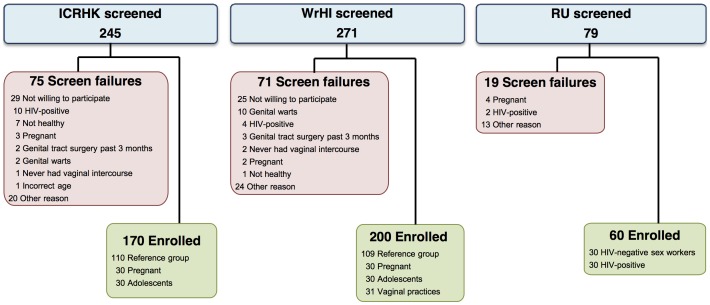
Recruitment flow chart. This Figure presents the screening and enrolment flow diagram, including reasons for screening failure. A total of 595 women were screened and 430 were enrolled in the study: 219 in the reference group; 60 pregnant women; 60 adolescents; 31 women engaging in vaginal practices; 30 FSW; and 30 HIV-positive women. ICRHK: International Centre of Reproductive Health, Mombasa, Kenya; RU: Rinda Ubuzima, Kigali, Rwanda; WrHI: Wits Reproductive Health and HIV Institute, Johannesburg, South Africa. Other ineligibility reasons: moving out of the area; not able to keep appointments due to mobility; unknown. Vaginal practices: Using traditional vaginal practices with anything other than water, soap and/or fingers alone.

A total of 595 women were screened and 430 were enrolled in the study: 219 in the reference group; 60 pregnant women; 60 adolescents; 31 women engaging in vaginal practices; 30 FSW; and 30 HIV-positive women. Figure 1 presents the screening and enrolment flow diagram, including reasons for screening failure. The median time between the screening and enrolment visits was 25 days (interquartile range 14–39 days).

### Sociodemographic, behavioural, and clinical characteristics

The mean ages were similar across the groups (25–26 years), except for the adolescents (16 years) and the HIV-positive women (30 years) (Table 1). Most adolescents and the majority of FSW and women in SA were not married. The educational level was highest in SA and lowest in Rwanda.

**Table 1:** Sociodemographic, behavioural, and clinical characteristics of the study population by group.

	Reference group		Pregnant women^1^		Adolescents		Vaginal practices^2^	Sex workers	HIV-positive^3^
	Kenya N = 110	South Africa N = 109	Kenya N = 30	South Africa N = 30	Kenya N = 30	South Africa N = 30	South Africa N = 31	Rwanda N = 30	Rwanda N = 30
	N (%)^4^	N (%)^4^	N (%)^4^	N (%)^4^	N (%)^4^	N (%)^4^	N (%)^4^	N (%)^4^	N (%)^4^
***Socio-demography***									
***Age in years***									
**Mean (range)**	25(18–35)	25(18–34)	26(19–34)	25(18–40)	16(16–17)	16(16–17)	24(19–33)	27(22–33)	31(22–35)
***Education***									
**None/some primary**	19(18)	3(3)	4(14)	0	7(23)	0	0	18(60)	9(30)
**Primary/some secondary**	52(48)	23(21)	10(34)	10(33)	19(63)	30(100)	12(39)	12(40)	10(33)
**Secondary/higher**	37(34)	84(76)	15(52)	20(67)	4(13)	0	19(61)	0	11(37)
***Marital status***									
**Never married**	29(32)	83(75)	2(7)	19(63)	28(93)	30(100)	27(87)	21(70)	5(17)
**Married**	62(57)	25(23)	28(93)	11(37)	2(7)	0	3(10)	0	18(60)
**Separated/divorced/widowed**	15(14)	2(2)	0	0	0	0	1(3)	9(30)	7(23)
***Living at current address***									
**0–12 months**	27(25)	25(23)	11(37)	11(37)	3(10)	2(7)	12(39)	2(7)	6(20)
**>12 months**	83(75)	84(77)	19(63)	19(63)	27(90)	28(93)	19(61)	28(93)	24(80)
***Sexual behaviour***									
***Age at first sexual intercourse***									
**Median (range)**	18(10–25)	18(13–25)	20(15–33)	19(15–23)	15(12–17)	16(13–17)	18(15–22)	16(13–23)	18(11–24)
***Lifetime number of sex partners***									
**1**	32(29)	18(17)	12(40)	6(20)	19(63)	18(60)	5(16)	0	2(7)
**2–3**	63(57)	50(46)	15(50)	17(57)	6(20)	11(37)	13(42)	0	16(53)
**>3**	15(14)	41(38)	3(10)	7(23)	5(17)	1(3)	13(42)	30(100)	12(40)
***Number of sex partners last 3 months*** 5									
**0**	9(8)	7(6)	0	1(3)	4(13)	2(7)	1(3)	0	2(7)
**1**	99(90)	99(91)	30(100)	28(93)	26(87)	25(83)	27(87)	1(3)	24(80)
**>1**	2(2)	3(3)	0	1(3)	0	3(10)	3(10)	29(97)	4(13)
***Sexual risk taking*** 6									
**Low**	57(52)	36(33)	24(80)	17(57)	10(33)	15(50)	9(29)	0	0
**Medium**	42(38)	59(54)	6(20)	12(40)	10(33)	12(40)	17(55)	0	0
**High**	11(10)	14(13)	0	1(3)	10(33)	3(10)	5(16)	30(100)	30(100)
***Frequency of sexual intercourse in the past 3 months***									
**No sexual intercourse**	19(17)	10(9)	0	1(3)	7(23)	12(40)	4(13)	0	2(7)
**< = 10 times**	32(29)	40(37)	6(20)	3(10)	14(47)	15(50)	12(40)	10(33)	8(27)
**11–30 times**	34(31)	37(34)	9(30)	12(40)	7(23)	1(3)	7(23)	13(44)	10(33)
**>30 times**	24(22)	19(17)	15(50)	14(47)	2(7)	1(3)	8(23)	7(24)	10(33)
***Reported recent vaginal sex at enrolment*** 7	19(17)	17(16)	10(33)	3(10)	2(7)	0	4(13)	16(53)	11(37)
***Seminal factor present at enrolment*** 8	25(23)	58(55)	16(53)	16(59)	5(17)	16(59)	14(45)	17(57)	16(53)
***Any sex partners within 3 months of enrolment***	91(83)	99(91)	30 (100)	29(97)	23(77)	18(60)	27(87)	30(100)	28(93)
***Condom use with last sexual contact*** 9	24(26)	38(38)	0	1(3)	16(70)	14(78)	11(41)	21(70)	17(61)
***Circumcision status partners*** 9: **All circumcised**	86(96)	53(59)	29(97)	12(43)	21(100)	9(56)	10(42)	9(30)	15(56)
***Wash inside vagina when bathing*** 10	66(60)	37(34)	11(37)	6(20)	13(43)	9(30)	28(90)	18(60)	13(43)
***Products used in vagina***									
**None**	35(32)	35(32)	17(57)	13(44)	16(54)	16(53)	0	10(33)	12(40)
**Water/fingers only**	26(24)	51(47)	3(10)	13(43)	4(13)	8(27)	0	17(57)	14(47)
**Water and soaps**	31(28)	23(21)	7(23)	2(7)	7(23)	4(13)	1(3)	3(10)	1(3)
**Cloth**	16(15)	0	2(7)	1(3)	2(7)	2(7)	23(74)	0	3(10)
**Lemon juice/detergents**	2(2)	0	1(3)	1(3)	1(3)	0	7(23)	0	0
***Reproductive health***									
***Parity***									
**0**	24(22)	34(31)	11(37)	20(67)	24(80)	27(90)	5(16)	1(3)	3(10)
**1–2**	62(56)	60(55)	15(50)	9(30)	6(20)	3(10)	24(78)	21(70)	17(57)
**>2**	24(22)	15(14)	4(13)	1(3)	0	0	2(6)	8(27)	10(33)
***Contraceptive use***									
**None**	25(23)	18(17)	Not Applicable		14(47)	7(23)	4(13)	2(7)	5(17)
**Condoms only**	24(22)	28(26)			12(40)	18(60)	11(35)	7(23)	9(30)
**Combined hormones**	20(18)	18(17)			1(3)	2(7)	5(16)	3(10)	2(7)
**Progestin-only injectable**	37(34)	42(39)			3(10)	3(10)	11(36)	18(60)	11(37)
**Intrauterine device**	4(4)	2(2)			0	0	0	0	3(10)
**Sterilisation**	0	1(1)			0	0	0	0	0
***Vaginal signs and symptoms***									
**Reported abnormal discharge**	4(4)	11(10)	0	6(20)	0	2(7)	1(3)	1(3)	0
**Vaginal discharge on speculum examination**	37(34)	14(13)	10(33)	4(13)	7(23)	5(17)	3(10)	8(27)	9(30)
***Degree of cervical ectopy***									
**Absent**	75(68)	46(43)	24(80)	19(63)	23(77)	18(60)	13(42)	6(20)	5(17)
**Small**	10(9)	15(14)	2(7)	1(3)	0	1(3)	5(16)	10(33)	10(33)
**Moderate**	22(20)	45(42)	4(13)	10(33)	7(23)	11(37)	13(42)	14(47)	15(50)
**Large**	3(3)	2(2)	0	0	0	0	0	0	0
***Reproductive tract infections at screening*** 11									
**Herpes simplex virus 2**	31(28)	44(40)	5(17)	11(37)	3(10)	1(3)	14(45)	14(47)	24(83)
**Syphilis**	0	0	0	1(3)	0	0	0	2(7)	6(20)
**Neisseria gonorrhoea**	1(1)	1(1)	0	0	0	0	1(3)	2(7)	4(13)
**Chlamydia trachomatis**	4(4)	18(17)	2(7)	4(13)	0	4(13)	8(26)	3(10)	0
**Trichomonas vaginalis**	3(3)	5(5)	1(3)	3(10)	4(13)	0	4(14)	3(10)	3(10)
**Candida species**	13(12)	29(27)	7(23)	17(57)	1(3)	11(37)	10(32)	3(10)	4(13)
***Nugent score at screening:*** ** Mean (SD)**	3.7(4.0)	3.4(4.1)	3.2(3.9)	3.8(4.4)	2.3(3.5)	5.3(4.4)	4.0(3.9)	4.7(3.8)	4.1(3.8)
***Bacterial vaginosis at screening***									
**Normal (Nugent 0–3)**	48(51)	60(59)	17(61)	16(55)	20(69)	11(38)	14(48)	10(37)	12(43)
**Intermediate (Nugent 4–6)**	12(13)	2(2)	2(7)	2(7)	3(10)	1(3)	5(17)	5(19)	6(21)
**Bacterial vaginosis (Nugent 7–10)**	34(36)	39(39)	9(32)	11(38)	6(21)	17(59)	10(34)	12(44)	10(36)
***Nugent score at enrolment:*** ** Mean (SD)**	3.6(4.0)	2.8(4.0)	2.6(3.9)	2.9(4.0)	2.4(3.6)	4.7(4.3)	3.1(3.9)	5.8(3.5)	4.5(4.3)
***Bacterial vaginosis at enrolment***									
**Normal (Nugent 0–3)**	55(55)	63(65)	19(68)	18(62)	18(67)	13(45)	16(59)	6(24)	13(48)
**Intermediate (Nugent 4–6)**	8(8)	6(6)	1(4)	2(7)	4(15)	4(14)	1(4)	2(8)	1(4)
**Bacterial vaginosis (Nugent 7–10)**	37(37)	28(29)	8(29)	9(31)	5(19)	12(41)	10(37)	17(68)	13(48)
**New systemic Antibiotic use 14 days before enrolment**	11(10)	19(17)	5(17)	7(23)	4(13)	4(13)	4(13)	4(13)	4(13)

1 Pregnant: up to 14 weeks gestational age at enrolment as defined by abdominal ultrasound;

2 Vaginal Practices: using other substances than the use of water, soap and/or fingers alone;

3 HIV-positive women: on antiretroviral treatment for at least 6 months, currently asymptomatic, and a CD4 count of more than 350 cells/µl.

4 N (%) of women with the characteristic; mean/median (range) when otherwise specified;

5 Last 3 months prior to screening visit;

6 Sexual risk taking: Low risk: 1 or no partners in last year AND did not have any partner (in the last 3 months) with multiple partners AND age first sex at least 15 years; Medium risk: 2 partners last year OR had at least one sexual partner (in the last 3 months) who had multiple partners; High risk: sex worker OR at least 3 partners last year OR at had at least one sexual partner with HIV in the last 3 months OR age first sex less than 15 years.

7 Recent vaginal sex: vaginal sex this morning and/or yesterday evening before enrolment visit;

8 Prostate specific antigen present in vaginal fluid, including weak reaction.

9 With partners in the 3 months prior to enrolment;

10 Most of the time/every time.

11 Data for *Trichomonas vaginalis* testing was unavailable for 8 women, HSV-2 for one, and Nugent scores slides were unreadable at screening for 36 and at enrolment for 41.

Age at first sexual intercourse was lower for the adolescents compared to the other groups, as sexual activity was an inclusion criterion for all women in the study. Kenyan women in the reference group, and adolescents in Kenya and SA, were less likely to have had three or more lifetime sex partners than women in the other groups (Table 1). Adolescents were less likely to report a (new or old) sex partner in the 3 months preceding the enrolment visit. The estimated frequency of sexual intercourse was highest for pregnant women and lowest for adolescents. FSW, HIV-positive women and adolescents reported the highest use of condoms.

Nineteen percent of women in the vaginal practices group used vaginal drying or tightening products before sex. Vaginal cleansing after sex was practiced by 90% of these women but was also a common practice among women in the other study groups. Vaginal washing as part of bathing was frequently reported by all groups although pregnant women reported somewhat less washing of the vagina. Women mostly inserted water and/or soap using their fingers, and women in the vaginal practices group also used cloth (74%) and lemon juice and/or detergents (23%). Nearly all women in Kenya reported that their partners of the past three months were circumcised compared to only half of the women in Rwanda and SA.

### Use of antibiotics

Systemic antibiotics, excluding treatment for opportunistic infection (26 women), were used by 62 women (14%), during the last 14 days before the enrolment visit. The last day of antibiotic use was on average 7 days (median 7 days) prior to the enrolment visit. Antibiotics were prescribed for 17 cases of BV (by Amsel criteria), 12 cases of *C. trachomatis*, 10 pelvic inflammatory disease cases, six cases of trichomoniasis, four cases of *N. gonorrhoea*, two syphilis infections, one ulcer disease infection, five respiratory infections, two urinary tract infections, and three other indications.

### Reproductive health characteristics

Use of contraceptives was lowest among adolescents. The most commonly used contraceptive methods were condoms and/or progestin-only injections but among adolescents the most used method was by far condoms (Table 1).

Unusual vaginal discharge on speculum examination was frequently observed despite the low reporting of vaginal discharge by the study participants themselves. At visual inspection with speculum examination only few large areas of ectopy were diagnosed, but moderate areas of ectopy were more common, in particular among FSW and HIV infected women.

The mean vaginal pH was 4.7 (standard deviation (SD)  = 0.7) with the majority (68%) of women having a vaginal pH between four and five. Ten percent of women had a pH below four (data not shown).

### Prevalence of reproductive tract infections

RTIs were common (Table 1), especially HSV-2 infection with prevalence rates ranging from 3% in South African adolescents to 83% in HIV-positive women. *T. vaginalis* and *C. trachomatis* prevalence rates were highest in the vaginal practices group (13% and 26%, respectively). *N. gonorrhoea* and syphilis were not common except in FSW (7% and 7%) and HIV-positive women (13% and 20%). *Candida* species were detected in 57% of the pregnant women in SA and 3–37% of the women in the other groups.

### Prevalence of BV by Nugent score

**Figure 2:**
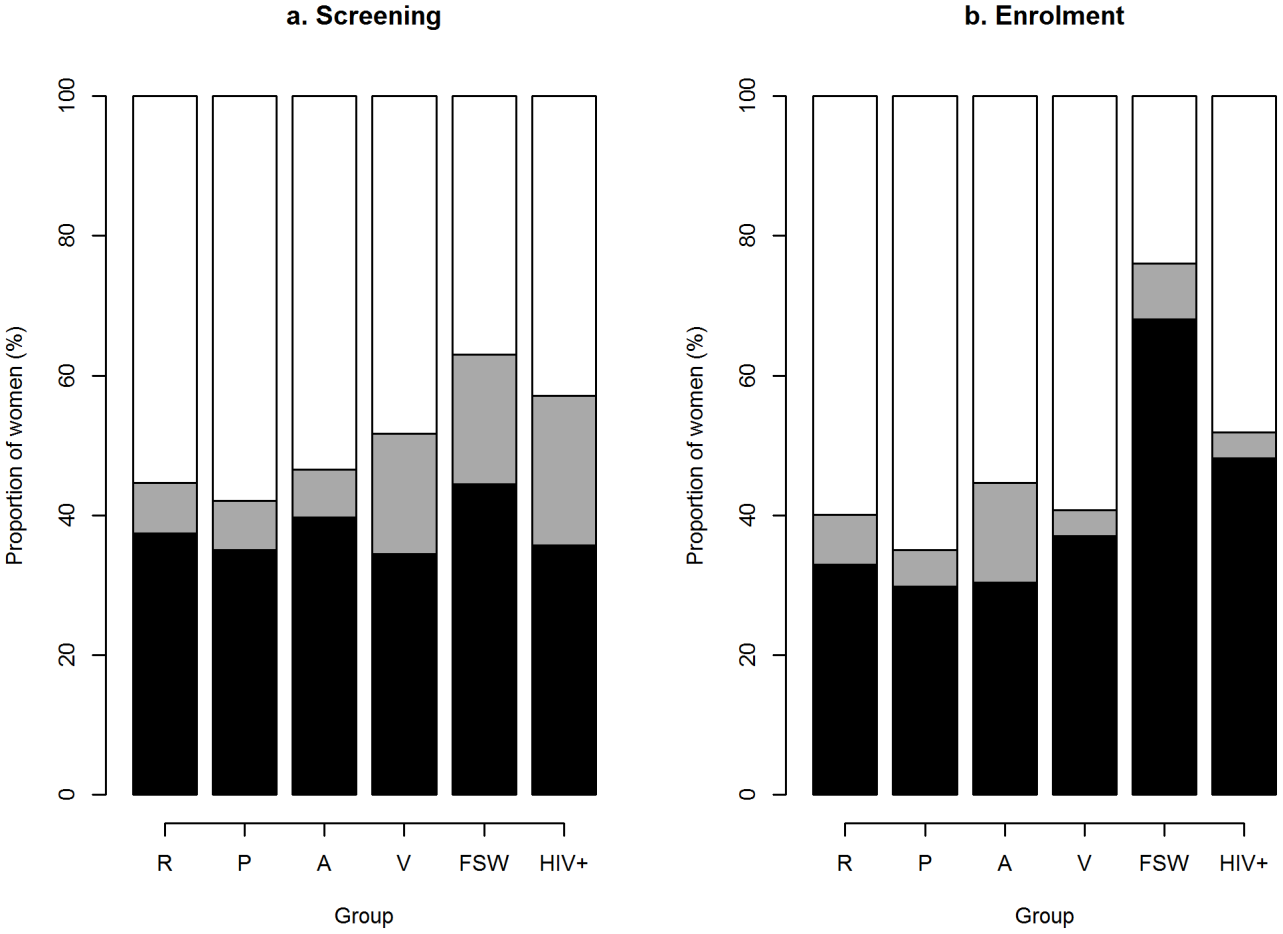
The prevalence of Bacterial Vaginosis at screening and at enrolment. The prevalence of BV at enrolment was highest in female sex workers (68%) and HIV-positive women (48%) in Rwanda. Black bar: Nugent score 7–10 classified as BV; Grey bar: Nugent score 4–6 classified as intermediate; White bar: Nugent score 0–3 classified as normal. Groups: R =  Reference group, P =  Pregnant women, A =  adolescents, V =  vaginal practices, FSW =  female sex worker, and HIV+ =  HIV-positive women.

Nugent scores were available for 394/430 (92%) women at screening and for 389/430 (90%) women at enrolment. For the remaining women, slides were unavailable (2/3, at screening/enrolment), inadequate (8/10) or could not be read due to the absence of bacteria (24/26) or presence of *Streptococcus* cell type (2/2). Of the scored smears, 38% (n = 148) had a Nugent score of 7–10 and 10% a Nugent score of 4–6 at screening. At enrolment, 36% (n = 139) had a Nugent score of 7–10 and 7% of 4–6. One in four of the BV scores at enrolment were incident cases of BV by Nugent score. The other 75% had BV by Nugent score at screening and at enrolment. The prevalence of BV at enrolment was highest in the FSW (68%) and HIV-positive women (48%) (Table 1 and Figure 2).

### Correlates of BV by Nugent score

Bivariable and multivariable logistic regression modelling of correlates of BV (Nugent score 7–10) at screening and at enrolment are presented in Table 2. At screening, the use of progestin-only contraception was associated with a lower prevalence of BV (OR 0.57; CI: 0.35–0.93), while the presence of more than one RTI was associated with a higher prevalence of a BV score (OR 5.07; CI: 1.32–19.5). Both associations remained statistically significant (p≤0.05) in the multivariable model.

**Table 2:** Correlates of bacterial vaginosis at the screening and enrolment visit - bivariable and multivariable models.

	BV^1^ at screening (37.6%)			BV^1^ at enrolment (35.7%)		
		Unadjusted	Adjusted^2^		Unadjusted	Adjusted
	N = 394	OR (CI)	OR (CI)	N = 389	OR (CI)	OR (CI)
Site	%	(p = 0.250)		%	(p = 0.002)	
**Kenya**	32.5	Ref.		31.8	Ref	
**Rwanda**	40.0	1.39 (0.73, 2.63)		57.7	2.92 (1.53, 5.58)	
**South Africa**	41.0	1.44 (0.92, 2.26)		32.0	1.01 (0.64, 1.60)	
**Age category**		(p = 0.337)			(p = 0.632)	
**<18 years**	39.7	Ref		30.4	Ref	
**18–24 years**	42.8	1.14 (0.61, 2.12)		36.5	1.32 (0.68, 2.57)	
**25–29 years**	32.3	0.73 (0.38, 1.38)		34.4	1.20 (0.61, 2.36)	
**30 years or more**	35.2	0.83 (0.40, 1.69)		41.2	1.61 (0.76, 3.39)	
**Marital Status**		(p = 0.196)			(p = 0.897)	
**Never married**	40.3	Ref		36.3	Ref	
**Married**	35.8	0.83 (0.53, 1.29)		34.3	0.92 (0.59, 1.43)	
**Sep/Div/Wid**	24.1	0.47 (0.19, 1.15)		37.9	1.07 (0.48, 2.38)	
**Education**		(P-value = 0.619)			(P-value = 0.670)	
**None/Some prim**	38.9	Ref		41.2	Ref	
**Prim/Some sec**	34.8	0.84 (0.44, 1.58)		35.5	0.79 (0.41, 1.50)	
**Sec/Higher**	39.8	1.04 (0.56, 1.94)		34.3	0.75 (0.39, 1.42)	
**Living at current address**		(p = 0.377)			(p = 0.423)	
**0–12 months**	41.6	Ref		39.3	Ref	
**>12 months**	36.4	0.80 (0.50, 1.30)		34.7	0.82 (0.50, 1.33)	
**Parity**		(p = 0.702)			(p = 0.535)	
**0**	39.9	Ref		32.1	Ref	
**1–2**	35.5	0.83 (0.53, 1.30)		37.9	1.29 (0.82, 2.04)	
**>2**	38.9	0.96 (0.51, 1.82)		37.0	1.24 (0.64, 2.39)	
**Early pregnancy** 3		(p = 0.675)			(p = 0.308)	
**No**	38.0	Ref		36.7	Ref	
**Yes**	35.1	0.88 (0.49, 1.59)		29.8	0.73 (0.40, 1.35)	
**Contraception**		**(p = 0.063)** 9	(p = 0.020)^9^		(p = 0.794)	
**None/Non-Horm**	41.1	Ref	Ref	37.0	Ref	
**Progesteron only**	28.6	0.57 (0.35, 0.93)	0.51 (0.31, 0.84)	33.3	0.85 (0.53, 1.37)	
**Combined Horm**	41.5	1.02 (0.52, 1.99)	1.06 (0.54, 2.09)	34.8	0.91 (0.47, 1.76)	
**Recent systemic antibiotic use** 4					**(p = 0.075)**	
**No**	NA			33.9	Ref	
**Yes**	NA			46.4	1.69 (0.95, 2.99)	
**Sex worker**		(p = 0.725)			(p≤0.001)	(p≤0.001)
**No**	37.3	Ref		33.3	Ref	Ref
**Yes**	40.7	1.15 (0.52, 2.56)		69.2	4.50 (1.90,10.64)	4.31 (1.79,10.39)
**Lifetime sex partners**		(p = 0.469)			**(p = 0.044)**	
**1**	38.8	Ref		25.7	Ref	
**2–3**	34.3	0.82 (0.50, 1.36)		38.4	1.80 (1.05, 3.09)	
**>3**	41.2	1.10 (0.64, 1.89)		40.5	1.97 (1.10, 3.53)	
**Sex partners last 3 months**		(p = 0.283)			**(p = 0.006)**	
**0**	22.7	Ref		16.7	Ref	
**1**	38.1	2.09 (0.75, 5.80)		34.9	2.68 (0.89, 8.02)	
**>1**	41.5	2.41 (0.74, 7.80)		55.3	6.18 (1.77, 21.55)	
**Condom use last sexual contact**		(p = 0.757)			(p = 0.345)	
**No**	37.1	Ref		34.3	Ref	
**Yes**	38.8	1.07 (0.69, 1.68)		39.4	1.25 (0.79, 1.96)	
**Circumcision status partners**		(p = 0.934)			(p = 0.291)	
**Not circumcised**	37.9	Ref		41.0	Ref	
**Circumcised**	37.4	0.98 (0.61–1.59)		34.8	0.77 (0.47–1.25)	
**Frequency of sexual intercourse in the past 3 months**		(p = 0.702)			(p = 0.373)	
**Up to 10 times**	39.1	Ref			Ref	
**11–30 times**	38.4	0.97 (0.59–1.61)			1.23 (0.73–2.06)	
**>30 times**	33.7	0.79 (0.45–1.40)			0.82 (0.46–1.44)	
**Recent vaginal sex at enrolment** 5					(p = 0.113)	
**No**	NA			33.9	Ref	
**Yes**	NA			43.8	1.52 (0.91, 2.56)	
**PSA present at enrolment** 6					**(p≤0.001)** 9	
**No**	NA			27.3	Ref	Ref
**Yes**	NA			47.3	2.39 (1.56, 3.66)	2.35 (1.52, 3.63)
**Wash inside vagina** 7		(p = 0.138)			(p = 0.418)	
**No**	42.9	Ref		39.4	Ref	
**Yes**	35.1	0.72 (0.47, 1.11)		30.8	0.68 (0.35, 1.34)	
**Yes, recently**		NA		33.9	0.79 (0.51–1.23)	
**Products used inside vagina**		(p = 0.416)			(p = 0.503)	
**None**	42.3	Ref		38.0	Ref	
**Water/fingers**	37.5	0.82 (0.50, 1.34)		38.7	1.03 (0.63, 1.70)	
**Water and soap**	31.8	0.64 (0.34, 1.18)		30.6	0.72 (0.39, 1.32)	
**Cloth/Substances**	32.8	0.67 (0.35, 1.26)		30.4	0.71 (0.37, 1.39)	
**HIV**		(p = 0.825)			(p = 0.177)	
**No**	37.8	Ref		35.0	Ref	
**Yes**	35.7	0.91 (0.41, 2.04)		48.1	1.72 (0.79, 3.78)	
**HSV-2**		(p = 0.504)			**(p = 0.086)**	
**No**	36.4	Ref		32.7	Ref	
**Yes**	39.8	1.16 (0.75, 1.78)		41.5	1.46 (0.95, 2.25)	
**Vaginal Candidiasis**		(p = 0.770)			**0.044**	
**No**	38.0	Ref		38.3	Ref	
**Yes**	36.3	0.93 (0.57, 1.51)		26.7	0.59 (0.35, 1.00)	
**RTIs** 8		**(p = 0.007)**	(p = 0.002)		**(p = 0.033)**	
**No RTIs**	34.5	Ref	Ref	33.0	Ref	
**1 RTI**	49.1	1.83 (1.03, 3.26)	1.89 (1.05, 3.38)	48.1	1.88 (1.04, 3.39)	
**>1 RTIs**	72.7	5.07 (1.32, 19.5)	6.80 (1.71, 27.1)	60.0	3.04 (0.84, 11.0)	

BV =  bacterial vaginosis; Horm  =  hormonal; HSV-2  =  herpes simplex virus type 2; NA =  not applicable; OR =  odds ratio; CI =  confidence interval; Prim  =  primary education; PSA  =  prostate specific antigen; Ref  =  reference category; RTI  =  reproductive tract infection; Sec  =  secondary education; Sep/Div/Wid  =  separated, divorced or widowed.

1 BV as defined by Nugent score: for the analysis we classified BV-Positive Nugent 7–10 versus BV-Negative Nugent 0–6;

2 Bolded p-values indicate significant associations at the level p≤0.100 for the bivariable analysis and were selected for multivariable modelling. Adjusted odd-ratios are only shown for factors which remained statistically significant at p≤0.050 level after stepwise deletion.

3 Early pregnancy: gestation less than 14 weeks;

4 Antibiotic use within the last 14 days;

5 Recent vaginal sex: vaginal sex this morning and/or yesterday evening (only recorded at enrolment visit);

6 Presence of PSA a marker of semen in vaginal fluid;

7 Wash vagina at screening: When you bathe, do you wash inside the vagina? Recently (only recorded at enrolment visit): this morning and/or yesterday evening;

8 Includes: syphilis, *Neisseria gonorrhoea*, *Chlamydia trachomatis*, *Trichomonas vaginali*s.

9 Associations remain statistically significant in the non-pregnant women group (N = 219).

At enrolment, the associations with being a self-acknowledged FSW (AOR 4.31; CI: 1.79–10.39) and recent unprotected sex as evidenced by the presence of PSA in vaginal fluid (AOR 2.35; CI: 1.52–3.63) were statistically significant (p≤0.05) in the multivariable model. Other factors that were associated with a BV score at p≤0.10 in bivariable analysis were: study site, recent antibiotic use, positive HSV-2 serology, and having more than one RTI (other than candidiasis, HIV or HSV-2 infection) at screening, having had more than one lifetime sex partner, and having had more than one sex partner in the past three months. FSW more often presented with incident BV by Nugent score at enrolment compared to the other women (AOR 4.69; CI: 1.44–15.2). No significant difference was seen for persistent BV by Nugent score at both visits between FSW and the other women. On the other hand, PSA presence was strongly related to persistent BV (AOR 2.22; CI: 1.36–3.62).

### Correlates of vaginal candidiasis and RTIs at screening

The prevalence of *Candida* species was higher in SA (adjusted odds ratio (AOR) 3.88; CI: 2.22–6.78) and in pregnant women (AOR 2.98, CI: 1.60–5.55) (Table 3). The likelihood of any RTI (syphilis, *N. gonorrhoea*, *C. trachomatis*, *T. vaginalis*) was lower when married (AOR 0.31; CI: 0.15–0.67) and when having lived at the same address for more than 12 months (AOR 0.51, CI: 0.27–0.98), but higher when engaging in vaginal practices (water/fingers AOR 2.87; CI 1.33–6.17; water and soap AOR 3.47; CI 1.37–8.78; cloth/substances AOR 3.34; CI: 1.33–8.38), the presence of ectopy (AOR 2.58; CI 1.39–4.80), positive HIV status (AOR 2.29; CI: 1.04–8.23), and vaginal dysbiosis (BV: AOR 3.14; CI: 1.66–5.94; intermediate microbiota: AOR 3.16; CI: 1.26–7.88). Positive HSV-2 serology was more common among women who were older than 30 (AOR 4.97; CI: 1.42–17.44), had given birth (1–2 children AOR 2.89; CI: 1.51–5.54; >2 children AOR 3.04; CI: 1.29–7.15), had had more than three lifetime sex partners (AOR 5.33; CI: 2.58–10.99), and were HIV infected (AOR 5.77; CI: 2.03–16.42). Women who had lived at the same address for more than 12 months were less likely to have positive HSV-2 serology (AOR 0.43; CI: 0.24–0.75).

**Table 3:** Correlates of reproductive tract infections at the screening visit - bivariable and multivariable models.

	Vaginal *Candida* 1 (22.1%)			Any of syphilis, *N. gonorrhoea, C. trachomatis* or *T. vaginalis* 2 (17.0%)			HSV-2 (34.3%)		
		Unadjusted^3^	Adjusted		Unadjusted^3^	Adjusted		Unadjusted^3^	Adjusted
	N = 430	OR (CI)	OR (CI)	N = 430	OR (CI)	OR (CI)	N = 429	OR (CI)	OR (CI)
Site	%	(p = <.001)^4^	(p = <.001)^4^	%	(p<.001)		%	(p<.001)	
**Kenya**	12.4	Ref	Ref	8.82	Ref		22.9	Ref	
**Rwanda**	11.7	0.94 (0.38, 2.33)	1.22 (0.48, 3.10)	26.7	3.76 (1.72, 8.20)		64.4	6.08 (3.20,11.55)	
**South Africa**	33.5	3.57 (2.08, 6.15)	3.88 (2.22, 6.78)	21.0	2.75 (1.46, 5.16)		35.0	1.81 (1.14, 2.87)	
**Age category**		(p = 0.944)			(p = 0.510)			**(p<.001)**	(p = 0.004)
**<18 years**	20.0	Ref		13.3	Ref		6.67	Ref	Ref
**18–24 years**	23.5	1.23 (0.59, 2.57)		20.1	1.64 (0.70, 3.82)		22.1	3.98 (1.34,11.79)	1.54 (0.48, 4.94)
**25–29 years**	22.1	1.14 (0.54, 2.40)		17.1	1.34 (0.57, 3.19)		43.6	10.81 (3.72,31.45)	2.93 (0.90, 9.54)
**30 or more**	21.0	1.06 (0.46, 2.43)		13.6	1.02 (0.38, 2.72)		61.3	22.13 (7.30,67.12)	4.97 (1.42,17.44)
**Marital Status**		(p = 0.254)			**(p = 0.002)** 4	(p = 0.005)^4^		**(p = 0.002)**	
**Never married**	23.6	Ref		21.1	Ref	Ref	28.0	Ref	
**Married**	22.0	0.91 (0.56, 1.49)		8.67	0.35 (0.19, 0.68)	0.31 (0.15, 0.67)	39.6	1.68 (1.09, 2.59)	
**Sep/Div/Wid**	11.8	0.43 (0.15, 1.28)		23.5	1.15 (0.49, 2.68)	0.85 (0.29, 2.50)	55.9	3.25 (1.56, 6.76)	
**Living at Current Address**		(p = 0.422)			**(p = 0.034)**	(p = 0.045)		**(p = 0.054)**	(p = 0.003)
**0–12 months**	19.2	Ref		24.2	Ref	Ref	42.4	Ref	Ref
**>12 months**	23.0	1.25 (0.72, 2.20)		14.8	0.54 (0.31, 0.94)	0.51 (0.27, 0.98)	31.8	0.63 (0.40, 1.00)	0.43 (0.24, 0.75)
**Parity**		(p = 0.104)			(p = 0.939)			**(p = <.001)** 4	(p = 0.004)
**0**	27.5	Ref		16.1	Ref		13.4	Ref	Ref
**1–2**	20.3	0.67 (0.41, 1.09)		17.5	1.11 (0.63, 1.94)		42.9	4.84 (2.81, 8.32)	2.89 (1.51, 5.54)
**>2**	15.6	0.49 (0.23, 1.05)		17.2	1.08 (0.49, 2.36)		54.0	7.56 (3.82,14.98)	3.04 (1.29, 7.15)
**Sex worker**		**(p = 0.073)**			(p = 0.356)			**(p = 0.066)**	
**No**	23.0	Ref		16.5	Ref		33.1	Ref	
**Yes**	10.0	0.37 (0.11, 1.25)		23.3	1.54 (0.63, 3.74)		50.0	2.02 (0.96, 4.26)	
**Lifetime sex partners**		(p = 0.423)			**(P-value = 0.035)**			**(p<.001)** 4	(p<.001)^4^
**1**	24.1	Ref		14.3	Ref		12.6	Ref	Ref
**2–3**	23.6	0.97 (0.56, 1.68)		13.6	0.95 (0.48, 1.85)		35.1	3.74 (1.99, 7.06)	2.76 (1.38, 5.52)
**>3**	18.1	0.70 (0.37, 1.30)		24.4	1.94 (1.00, 3.77)		52.0	7.50 (3.88,14.50)	5.33 (2.58,10.99)
**Products used inside vagina**		(p = 0.113)			**(p = 0.004)** 5	(p = 0.009)^4^		**(p = 0.012)**	
**None**	22.1	Ref		9.09	Ref	Re	25.5	Ref	
**Water/fingers**	16.9	0.72 (0.40, 1.29)		23.5	3.08 (1.56, 6.06)	2.87 (1.33, 6.17)	42.6	2.17 (1.32, 3.58)	
**Water and soap**	22.8	1.04 (0.54, 1.99)		16.5	1.97 (0.88, 4.43)	3.47 (1.37, 8.78)	31.6	1.35 (0.74, 2.46)	
**Cloth/Substances**	32.8	1.72 (0.89, 3.32)		23.0	2.98 (1.32, 6.70)	3.34 (1.33, 8.38)	41.0	2.03 (1.08, 3.80)	
**Early pregnancy** 5		**(p<.001)**	(p<.001)		(p = 0.945)			(p = 0.174)	
**No**	19.2	Ref	Ref	17.0	Ref		35.5	Ref	
**Yes**	40.0	2.81 (1.58, 5.00)	2.98 (1.60, 5.55)	16.7	0.97 (0.47, 2.02)		26.7	0.66 (0.36, 1.22)	
**HIV**		(p = 0.212)			**(p = 0.069)**	(p = 0.047)		**(p<.001)**	(p<.001)
**No**	22.6	Ref		16.1	Ref	Ref	30.9	Ref	Ref
**Yes**	13.3	0.53 (0.18, 1.55)		30.0	2.24 (0.98, 5.11)	2.92 (1.04, 8.23)	24/29 (82.8)	10.73 (4.00,28.79)	5.77 (2.03,16.42)
**BV**		(p = 0.883)			**(p<.001)**	(p<.001)		**(p = 0.093)**	
**Negative (0–3)**	24.0	Ref		9.62	Ref	Ref	29.8	Ref	
**Intermediate (4–6)**	21.1	0.84 (0.36, 1.96)		28.9	3.83 (1.65, 8.86)	3.16 (1.26, 7.88)	47.4	2.12 (1.05, 4.28)	
**Positive (7–10)**	22.3	0.91 (0.55, 1.50)		23.6	2.91 (1.60, 5.29)	3.14 (1.66, 5.94)	35.8	1.31 (0.84, 2.06)	
**Ectopy of the cervix** 6		(p = 0.316)			**(p = 0.002)**	(p = 0.002)		**(p = 0.009)**	
**No**	24.0	Ref		11.8	Ref	Ref	28.5	Ref	
**Yes**	20.0	0.79 (0.50, 1.25)		23.0	2.23 (1.33, 3.76)	2.58 (1.39, 4.80)	40.5	1.71 (1.14, 2.55)	

BV =  bacterial vaginosis; HSV-2  =  herpes simplex virus; PSA  =  prostate specific antigen; Ref  =  reference category; RTI  =  reproductive tract infection; Sep/Div/Wid  =  separated, divorced, or widowed.

1 Vaginal *Candida* as diagnosed by wet mount microscopy;

2 87 infections in 73 women;

3 Bolded p-values indicate significant associations at the level p≤0.100 in the bivariable analysis and were selected for multivariable modelling. Adjusted odd-ratios are only shown for factors which remained statistically significant at p≤0.050 level after stepwise deletion.

4 Associations remained statistically significant in the non-pregnant women group (N = 219).

5 Early pregnancy: gestation less than 14 weeks;

6 Ectopy  =  cervical ectopy; clinically observed and defined as No  =  absent, and Yes  =  small, moderate or large. OR: odds ratio; CI: confidence interval.

## Discussion

The main objective of our project was to characterise the vaginal environment in a reference group of women and in different groups of African women who would be recruited in trials of biomedical interventions or would be targeted for such interventions. As such we recruited women through reproductive health services and from selected communities. In most groups of women the prevalence of BV was in the range of 30 to 40%, except for adolescent girls who had a lower prevalence and FSW and HIV infected women who had a higher prevalence. There are some data available on the prevalence of BV in similar populations and sites but comparing prevalence rates is challenging. Previous studies conducted among pregnant women in KwaZulu Natal (South-Africa) found prevalence rates of over 50% [8] which is substantially higher than in our study but it should be noted that we excluded HIV infected pregnant women. Among a sample of 40 adult women attending a perinatal clinic in Johannesburg 35% were found to have BV as assessed by Nugent score which is in line with our findings [9]. Among FSW in Mombasa the prevalence of BV was lower than among the FSW in Kigali in our study [10]. Possible explanations for this difference could be more intensive follow-up of the FSW in Mombasa with frequent treatment and/or the fact that men in Mombasa are more frequently circumcised than men in Kigali [10].

At the screening visit, i.e. before any antibiotic treatment for symptomatic BV, RTIs or other infections were given, BV was associated in a multivariable analysis with the presence of more than one other RTI (syphilis, trichomoniasis, gonorrhoea, chlamydial infection) and with the use of progestin-only contraception. As this was a cross-sectional study it is not possible to ascertain the temporal relationship between BV and other RTIs. There is some evidence that other RTIs are risk factors for BV [11] and that BV enhances the vulnerability of women to other RTIs [12]–[14]. Our finding of an association between BV and use of progestin-only contraception is in agreement with two longitudinal studies that found hormonal contraception to be protective against BV [15], [16].

At the enrolment visit BV was associated with being a sex worker and with having had sexual intercourse recently, as evidenced by the presence of PSA in cervicovaginal fluid.

The presence of PSA is indicative of unprotected sexual intercourse within 14 up to 72 hours before sampling. The strong association we found between BV and presence of PSA adds to the evidence that exposure to vaginal penetration and male seminal plasma deposition contributes to the development and sustainment of BV. In a study of a large series of Papanicolaou smears taken before and after unprotected coitus, it was observed that lactobacilli-dominated precoital smears were replaced by *G. vaginalis* dominant microbiota in postcoital smears [17]. Another study showed that having had sex within the past 24 hours compared to more than a week ago increased the Nugent score significantly [18]. The most straightforward explanation for this is that unprotected sexual intercourse alters the physico-chemical vaginal environment thereby also affecting the vaginal microbiota.

At enrolment being a FSW was strongly related to having a BV score. Further, several sexual behavioural factors were associated in bivariable analysis: having had more than one lifetime sex partner, having had more than one sex partner in the past three months, positive HSV-2 serology, and having more than one RTI (other than candidiasis, HIV or HSV-2 infection) at screening. The fact that a BV score was associated with FSW status at enrollment, but not at screening may be a chance finding or alternatively at screening the contrast between FSW and other women may be smaller because of untreated RTIs in the non-FSW population. As FSW are more likely to seek treatment on a more regular basis. It may also be partially due to the significant higher number of new cases of incident BV in the FSW group compared to the other women. New RTI infections and the high HSV-2 prevalence in this highly sexually active group could have led to a higher incidence of BV. As we did not routinely retest for RTIs at the enrolment we can't exclude new infections at this visit which may have caused shifts in microbiota composition.

In our study we could not find a statistically significant association between BV and a number of sexual behavioural risk factors that were documented in other studies, including higher numbers of lifetime sex partners, lack of condom use and frequency of sexual intercourse [19]. There was also hardly any difference in prevalence of BV between women who reported that their male partners were circumcised compared to women who had sexual intercourse with uncircumcised men whereas Gray et al found that circumcision of male partners is protective against BV [10]. We attribute this to the low power of our study. Although the overall sample size was high, the sample size in each sub-population was rather low.

We did not find vaginal washing to be associated with BV, neither at screening nor at enrolment. Some studies have found douching and vaginal practices to be associated with BV but others could not confirm this [20]. Interestingly we found an association between vaginal washing and other RTIs. However it is not clear whether vaginal washing increases the susceptibility to these RTIs or whether women more frequently wash the vagina inside when they become symptomatic with RTIs.

We encountered several limitations in the study. An important limitation in the analysis was the use of data from the screening visit for the analysis at enrolment. The following data was collected at screening: RTIs (CT, NG, TV, HSV-2 and syphilis), which were not re-tested at enrolment, and the data of number of lifetime sex partners and number of sex partners in the past three months. Still, we believe that the main finding of the paper, the association of PSA and BV, is not affected by this shortcoming. For the laboratory testing, some cases of candidiasis may have been missed because diagnostic laboratory testing for *Candida* relied on microscopic evaluation and did not include culture. The diagnosis of *T. vaginalis* was performed by InPouch culture instead of nucleic acid amplification testing, which was not available to us at the time of our study. However, the sensitivity of InPouch culture when the culture is assessed after five days is 94% compared to nucleic acid amplification testing.

In conclusion, our study confirmed some of the correlates of BV that have been found in other studies but the most salient finding was the association between BV and the presence of PSA in the cervicovaginal fluid. A detailed characterisation of the vaginal microbiota will allow us to explore this association in more detail.

## Supporting Information

Table S1 **Dataset.**
(XLS)

Methods S1 **Study flowchart – evaluations.** This chart describes the cohort study with visit flow and evaluations. Women were followed up every two weeks for a total of four visits over two menstrual cycles (visits 2–5), and again three (visit 6) and six months later (visit 7). Visits 3, 5, 6 and 7 coincided approximately with day 9 of the menstrual cycle and visits 2 and 4 with day 23. For women without a menstrual cycle, the same time structure was followed.
(DOCX)
