# Supplementary material for: Prevalence and Correlates of Bacterial Vaginosis in Different Sub-Populations of Women in Sub-Saharan Africa: A Cross-Sectional Study
Source: PLoS One. 2014 Oct 7;9(10):e109670. doi: 10.1371/journal.pone.0109670 (PMC4188821; doi:10.1371/journal.pone.0109670)
Supplement: Methods S1 — Study flowchart – evaluations. This chart describes the cohort study with visit flow and evaluations. Women were followed up every two weeks for a total of four visits over two menstrual cycles (visits 2–5), and again three (visit 6) and six months later (visit 7). Visits 3, 5, 6 and 7 coincided approximately with day 9 of the menstrual cycle and visits 2 and 4 with day 23. For women without a menstrual cycle, the same time structure was followed. (DOCX) [file pone.0109670.s002.docx]

**Supporting information**

**Methods S1 Study flowchart - evaluations**

| **Clinic visit** | **Screening** | | **Enrolment**  **Visit 1**  **Day 9** | | **Visit 2**  **Day 23** | **Visit 3**  **Day 9** | **Visit 4**  **Day 23** | **Visit 5**  **Day 9** | **Visit 6**  **+ 3 months** | **Visit 7**  **+ 6 months** |
| --- | --- | --- | --- | --- | --- | --- | --- | --- | --- | --- |
| **Diagnostic tests** | |  | |  |  |  |  |  |  |  |
| *Chlamydia trachomatis* | | **x** | |  |  |  |  | **x** |  | **x** |
| *Neisseria gonorrhoea* | | **x** | |  |  |  |  | **x** |  | **x** |
| *Trichomonas vaginalis* | | **x** | |  |  |  |  | **x** |  | **x** |
| Vaginal candidiasis | | **x** | | **x ¹** | **x ¹** | **x ¹** | **x ¹** | **x ¹** | **x ¹** | **x ¹** |
| Pap smear | | **x** | |  |  |  |  |  |  | **◊** |
| Syphilis serology | | **x** | |  |  |  |  | **x** |  | **x** |
| HIV-serology | | **x** | |  |  |  |  | **x** |  | **x** |
| HSV-2 serology | | **x** | |  |  |  |  | **x** |  | **x** |
| Urine pregnancy test | | **x** | | **x** |  | **x ¹** |  | **x ¹** | **x ¹** | **x** |
| Urine dipstick test | | **x** | | **x** | **x ¹** | **x ¹** | **x ¹** | **x ¹** | **x** | **x ¹** |
| **Vaginal microbiota** | |  | |  |  |  |  |  |  |  |
| pH with paper strip | | **x** | | **x** | **x** | **x** | **x** | **x** | **x** | **x** |
| Amsel criteria | | **x** | |  |  |  |  |  |  |  |
| Nugent score by Gram stain microscopy | | **x** | | **x** | **x** | **x** | **x** | **x** | **x** | **x** |
| Culture of vaginal bacteria for H_2_O_2_ production | |  | | **x** |  |  |  | **x** |  |  |
| Quantification of Lactobacilli and BV associated bacteria by qPCR | |  | | **x** | **x** | **x** | **x** | **x** |  | **x** |
| **Immune activation (non-diagnostic)** | |  | |  |  |  |  |  |  |  |
| Soluble markers | |  | | **x** | **x** | **x** | **x** | **x** |  | **x** |
| Prostate Specific Antigen | |  | | **x** | **x** | **x** | **x** | **x** |  | **x** |

¹: performed only when indicated

Women were followed up every two weeks for a total of four visits over two menstrual cycles (visits 2-5), and again three (visit 6) and six months later (visit 7). Visits 3, 5, 6 and 7 coincided approximately with day 9 of the menstrual cycle and visits 2 and 4 with day 23. For women without a menstrual cycle, the same time structure was followed.
